# Postpyloric decompression tube placement through a gastrostomy for malignant bowel obstruction

**DOI:** 10.1186/1756-0500-6-217

**Published:** 2013-06-03

**Authors:** Yusuke Kurita, Tomoko Koide, Seitaro Watanabe, Tatsuya Ogawa, Yusuke Sekino, Hiroshi Iida, Takashi Nonaka, Akihiko Kusakabe, Eiji Gotoh, Shin Maeda, Atsushi Nakajima, Masahiko Inamori

**Affiliations:** 1 Gastroenterology Division, Yokohama City University Hospital, 3-9 Fukuura, Kanazawa-ku, Yokohama 236-0004, Japan; 2 Department of Medical Education, Yokohama City University School of Medicine, Yokohama, Japan

**Keywords:** Malignant bowel obstruction, Gastrostomy, Palliative care, Quality of life

## Abstract

**Background:**

Malignant bowel obstruction affect a patient’s quality of life, but, management of MBO is controversial.

**Case presentation:**

A 51-year-old woman who had been diagnosed as uterine cervix cancer 2 years ago and had undergone surgery, chemotherapy and radiotherapy, was admitted to our hospital. She was diagnosed as having a recurrence of peritoneal metastasis and bowel obstruction. For her nasal pain, we considered insertion of a postpyloric decompression tube through the gastrostomy instead of via the nasal cavity. After insertion of a percutaneous gastrostomy tube was performed endoscopically, we introduced a postpyloric decompression tube through her gastrostomy. She could be discharged home, and 91 days later, she died in her home under hospice care, as she had wished.

**Conclusions:**

Insertion of a postpyloric decompression tube through a gastrostomy might be useful in the management of advanced cancer patients with bowel obstruction.

## Background

Malignant bowel obstruction (MBO), a common complication in patients with advanced cancer, can significantly affect a patient’s quality of life [1-3]. However, management of MBO is controversial [4-6].

## Case presentation

A 51-year-old woman who had been diagnosed as having stage 2b uterine cervix cancer 2 years ago and had undergone surgery, chemotherapy and radiotherapy, was admitted to our hospital with nausea and abdominal pain. She was diagnosed as having a recurrence of peritoneal metastasis with complicating ascites and bowel obstruction. We first treated her conservatively however, a month later, her symptoms recurred and a postpyloric decompression tube was introduced via the nasal cavity. After the procedure, she complained of severe nasal pain and expressed her wish for treatment by a different method.

**Figure 1:**
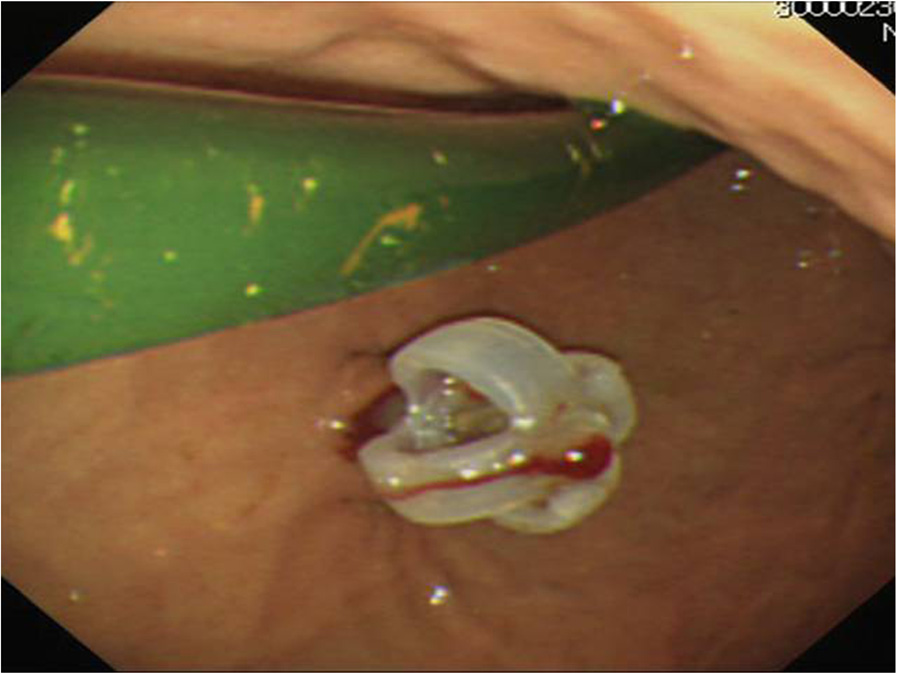
Percutaneous gastrostomy tube insertion was performed endoscopically, while a transnasal postpyloric decompression tube was present in her stomach.

**Figure 2:**
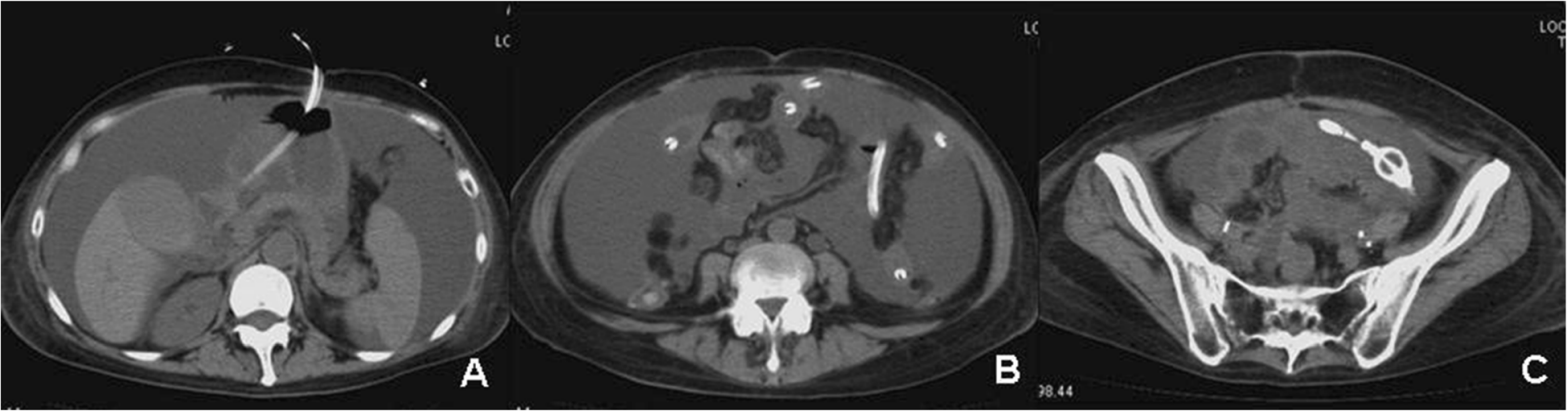
Postpyloric decompression tube was inserted through the gastrostomy.

We therefore considered insertion of a postpyloric decompression tube through the gastrostomy instead of via the nasal cavity. Following obtainment of informed consent, insertion of a percutaneous gastrostomy tube was performed endoscopically (Figure 1). Two weeks later, we introduced a postpyloric decompression tube through her gastrostomy instead of via the nasal cavity. The postpyloric decompression was effective (Figure 2), she was discharged home, and 91 days later, she died in her home under hospice care, as she had wished.

## Conclusions

Palliation of symptoms is the treatment goal terminal disease patients with MBO. Hospitalization and conservative management by nasogastric tube decompression and bowel rest is the first step in the management of MBO. However, when continuous postpyloric decompression is required, insertion of the postpyloric decompression tube through the gastrostomy instead of via the nasal cavity may be a reasonable approach, especially in patients hesitating to undergo tube placement via the conventional nasal approach and/or in gynecologic malignancies patients who are not good surgical candidates.

In conclusion, insertion of a postpyloric decompression tube through a gastrostomy might be useful in the management of advanced cancer patients with bowel obstruction.

## Consent

Written informed consent was obtained from the patient and her family for publication of this Case report and any accompanying images. A copy of the written consent is available for review by the Editor of this journal.

## Competing interests

The authors declare that they have no competing interest.

## Authors’ contributions

TK, AK and MI gave information about gastrostomy. SW and MI collect clinical data. YS, HI and TN gave information about endoscopy. AK gave information about palliative care. YK, TO and MI wrote the manuscript. EG, SM and AN supervised the manuscript. All authors read and approved the final manuscript.
